# Lactate increases oxygen unloading of preconditioned blood from male elite breath‐hold divers

**DOI:** 10.14814/phy2.70698

**Published:** 2026-01-07

**Authors:** Thomas Kjeld, Egon Godthaab Hansen, Henrik Christian Arendrup, Jens Højberg, Anders Nedergaard, Thomas Krag, John Vissing

**Affiliations:** ^1^ Copenhagen Neuromuscular Center Rigshospitalet, University of Copenhagen Copenhagen Denmark; ^2^ Department of Anesthesiology Herlev Hospital, Herlev, University of Copenhagen Copenhagen Denmark; ^3^ Department of Clinical Medicine, Faculty of Medicine University of Copenhagen Copenhagen Denmark; ^4^ Department of Cardiothoracic Anesthesiology Rigshospitalet, Copenhagen, University of Copenhagen Copenhagen Denmark

**Keywords:** erythorcyte metabolism, freediving, heart metabolism, Hypoxia, lactate

## Abstract

Physical performance can be improved in aerobic athletes and breath‐hold divers (BHD) by limb exposure to repetitive ischemia: remote ischemic conditioning (RIC). RIC protects against cardiac ischemia, and its blood‐borne transferable substrate could be lactate. Accordingly, lactate added to whale blood increases oxygen unloading and adult seals possess higher cardiac lactate dehydrogenase activity (LDHa) than terrestrial mammals. Because BHD and adult diving mammals share adaptations to hypoxia, including lactate metabolization during apnea, we hypothesized that BHD compared to BMI/VO_2_max‐matched aerobic controls have higher LDHa and lactate added to blood from BHD unloads oxygen more efficiently. Six BHD and six matched aerobic controls underwent RIC: three cycles of 5‐min inflation and 4‐min deflation of a blood pressure cuff on the dominant arm, maximum apnea after three submaximal apneas (BHD only), and a VO_2_max‐test. Blood‐samples were collected from the nondominant radial artery and the vena basilica of the dominant arm at rest, before termination of the three interventions, and for LDHa. Blood gases were compared to samples added lactate or placebo suspension. BHD had ⁓30% higher cardiac/erythrocyte LDHa compared to controls (*p* < 0.05). Lactate added to arterial blood from BHD after RIC increased oxygen unloading (*p* < 0.05). PaO_2_ decreased ⁓66% during apnea (375+/−49 s; *p* < 0.001; BHD only). We conclude that 1 (erythrocyte‐ and cardiac‐LDHa is higher in BHD compared to matched controls, and 2) lactate facilitates oxygen‐unloading in blood from BHD after RIC, similar to diving mammals.

## 1 INTRODUCTION

Remote ischemic conditioning (RIC), exposing, for example, an arm to repetitive ischemia and reperfusion is not only reported to be cardioprotective in selected patient populations (Botker et al., 2010) but also enhances performance in both highly trained aerobic athletes and anaerobic athletes like elite breath‐hold divers (BHD) (Crisafulli et al., 2011; de Groot et al., 2010; Jean‐St‐Michel et al., 2011; Kjeld et al., 2014; Patterson et al., 2015). The mechanisms underlying RIC are still not understood but seem to involve neurogenic as well as blood‐borne humoral factors (Lassen et al., 2021) that may synergistically mediate protection against ischemic or hypoxic exposure and also performance‐improving capacity (Dave et al., 2008; Endres, Fan, et al., 2000; Endres, Meisel, et al., 2000). Further mechanisms including derivatives of the hypoxic exposure during RIC, i.e. lactate likely contribute (Koyama, 2023). Lactate is the end product of anerobic metabolism, and it can be catalyzed to pyruvate by Lactate dehydrogenase (LDH). LDH has five isomeric forms: LDH‐1 is expressed preferentially in the heart and red blood cells; LDH‐2 in the reticuloendothelial system and red blood cells; LDH‐3 in the lungs; LDH‐4 in the kidneys; and LDH‐5 in the liver and skeletal muscle (Khan et al., 2020). Lactate is considered a protective agent because it increases recovery following strenuous exercise or during reperfusion like RIC following severe ischemia (Koyama, 2023). Lactate may be involved in the cardioprotective process of RIC because lactate‐enriched blood reduces lethal reperfusion injury more than RIC in humans (Koyama, 2023). Production of lactate is a metabolic consequence of both exercise and hypoxia, and blood from a preconditioned subject therefore contains lactate. Similarly, when a whale dives repeatedly, and hence by diving is exposed to repetitive hypoxia similar to RIC, its blood contains lactate and may contain the effects of RIC as mentioned above. Accordingly, in vitro added lactate to whole blood from a freshly hunted whale (Balaenoptera acutorostrata) increases the oxygen unloading by as much as 30%, indicating erythrocyte adaptations towards hypoxia (Brix et al., 1990). Similarly, the heart of adult harbor seals (Phoca vitulina) possesses higher LDH activity compared to terrestrial animals indicating cardiac metabolic adaptations to hypoxia (Fuson et al., 2003). Lactate could therefore be the substrate for eliciting the effects of RIC assuming that the cardioprotective and performance‐improving effect of RIC is unloading oxygen and facilitated by lactate. However, preconditioning may only be elicited in individuals with adaptations to hypoxia like adult diving mammals and BHD, but unlike seal pups and matched aerobic controls (Kjeld et al., 2018; Kjeld, Moller, et al., 2021). We therefore suggest that erythrocyte and cardiac LDH activity is higher in BHD as compared to matched controls and that adding lactate to blood from BHD unloads oxygen more efficiently than added to blood from matched controls. To test these hypotheses, we examined the LDH activity and effect of lactate added in vitro to blood from BHD and controls matched by BMI and maximal oxygen uptake (VO_2_max) (1) at rest, (2) after preconditioning, (3) after maximum apnea, and (4) after maximum aerobic exercise on a cycle ergometer.

## 2 METHODS

This research involving human participants has been approved by the Regional Ethics Committee of Copenhagen (H‐1‐2013‐060.). All clinical investigations have been conducted according to the principles expressed in the Declaration of Helsinki. Informed consent, written and oral, have been obtained from the participants.

Twelve healthy male nonsmoking subjects participated in the study (Table 1). Six subjects were elite BHD; age 42 ± 8 years and static apnea personal records >4 min (Joulia et al., 2015). For comparison 6 black belt judo athletes were chosen as they are aerobic athletes and considered elite within their sport with highest level of achievement (Black Belt). Furthermore, the duration of a judo match (4 min) is similar to the inclusion criteria for the minimum duration of static apnea for the BHD. The subjects were matched according to morphometric variables and whole‐body aerobic capacity (VO_2_max). Only male subjects were included, to diminish differences caused by gender in this limited number of subjects.

**TABLE 1:** Subject characteristics.

	BHD	Controls
*N*	6 males	6 males
Age (years)	45 ± 5	34 ± 11
Static breath‐hold personal best (seconds)	352 ± 44	N/A
Height (cm)	191 ± 7	182 ± 3*
Weight (kg)	83 ± 7	82 ± 10
Body Mass (kg/m^2^)	22.8 ± 3.1	24.6 ± 2.2
Maximal oxygen uptake, VO_2_ max (mL O_2_/min/kg)	52.7 ± 8.2	58.2 ± 5.9
Hemoglobin concentration (mmol/L)	9.7 ± 0.5	9.1 ± 0.1

*Note*: Basic morphometric data. Values are mean ± standard deviation.

Abbreviation: BHD, breath‐hold divers.

* *p* = 0.013.

The breath‐hold divers ranked among national top 10, three ranked among World top 10 and one was a World record holder.

**FIGURE 1:**
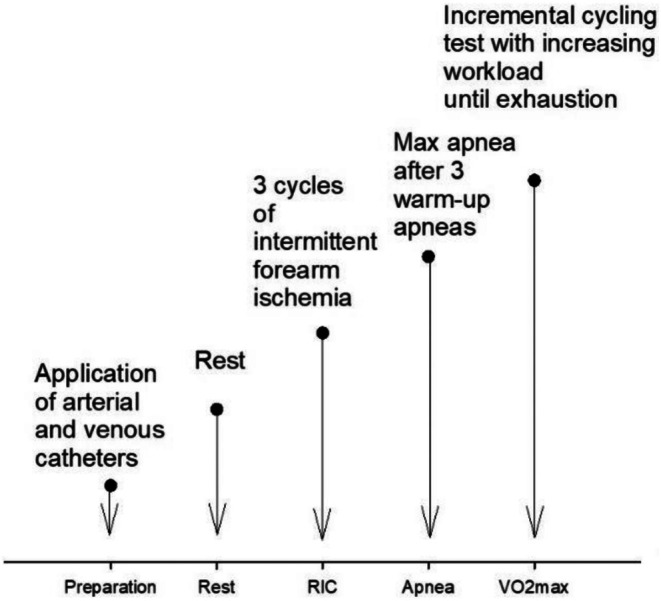
Time course for the study. RIC, remote ischemic preconditioning.

The following were the procedures during the study:

1. *At rest*, an arterial 1.1 mm, 20 gauge catheter was inserted in the radial artery of the nondominant arm and connected to a transducer for continuous flow of saline (Baxter, Uden, the Netherlands). Also, a catheter with same gauge as the above was placed in the vena basilica of the dominant arm.
2. Subjects were then exposed to RIC by intermittent forearm ischemia through three cycles of 5‐min inflation (to 40 mmHg above palpatory systolic blood pressure) and 4‐min deflation of a cuff (Wellch‐Allyn Durashock sphygmomanometer, NY) on the dominant arm (Botker et al., 2010; Kjeld et al., 2014). The below mentioned activity started within 15 min after intermittent arm ischemia:
3. BHD then performed maximum apnea after a warm‐up of three consecutive apneas with 4 min pause in between. Judo athletes perform aerobic sport, and thus did not perform apneas in the study, because they do not train apneas.
4. All subjects then completed a standardized warm‐up followed by an incremental cycling test starting at a workload of 100 W and increased by 50 W every minute until voluntary exhaustion (Figure 1): VO_2_max was defined as the highest recorded 30 s average oxygen uptake (VO_2_) during the test. For recognition of true VO_2_max, the following criteria had to be met: individual perception of exhaustion according to the Borg scale (20), heart rate approaching age‐predicted maximum (BHD 183 ± 12 bpm; controls 198 ± 25 bpm), and inability to maintain a pedaling frequency above 70 rpm (Table 1). Heart rate was recorded using a Lifepack® 20 monitor (Physio‐Control, Redmond USA).

Three blood samples were obtained from both the arterial and venous catheter after each of the abovementioned procedures. Venous blood samples were then added to either an L‐lactate solution (100 mM sodium lactate in 0.9% NaCl solution added 1:20 to 1 mL of blood for a final concentration of 5 mM lactate) (Kuze et al., 1992), a placebo solution (0.9% NaCl solution) or no supplement. The arterial blood samples were prepared in the same way.

LDH isoenzyme activity was analyzed using the Hydragel ISO‐LDH kit according to the manufacturer's instructions (SEBIA, Evry, France).

Blood gas analyses were performed immediately after sampling using an automated self‐calibrating blood gas machine (ABL 725 and ABL 90 Flex, Radiometer, Copenhagen, Denmark) for evaluation of pH, bicarbonate, lactate, PaCO_2_, and the arterial oxygen tension (PaO_2_). Lactate solution (100 mM sodium L‐lactate in 0.9% saline solution, Sigma‐Aldrich, St. Louis, MO) was added to respectively arterial and venous blood samples for a final concentration of 5 mM lactate, 12.7 mM Na^+^, and 7.7 mM Cl^−^. p50 was calculated using the Siggaard‐Andersen equation with pH, sO_2_, pO_2_, and bicarbonate (HCO_3_−) as factors (Aberman et al., 1975):

$${\mathrm{sO}}_{2}=100\times \left(\frac{X^{3}+150X}{X^{3}+150X+23400}\right)$$

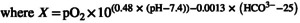

$$p50=\frac{{\mathrm{pO}}_{2}\times 10^{\left(\mathrm{pH}-\mathrm{pKa}\right)}}{{\mathrm{sO}}_{2}\times \frac{1-0.28\times {\mathrm{HCO}}^{3-}}{0.03\times {\mathrm{pCO}}^{2}}}$$

In the equation, p50 represents the estimated partial pressure of oxygen (_2_pO_2_) at which hemoglobin is 50% saturated, and pKa is the negative logarithm of the acid dissociation constant of the Henderson‐Hasselbalch equation for the bicarbonate buffer system (assumed to be 6.1).

### 2.1 Statistics

Variables are presented as mean ± standard deviation (SD). Data were analyzed using one‐way ANOVA repeated measures. The Holm‐Sidak method was used to evaluate differences between the two groups of subjects. A *p* value <0.05 was considered statistically significant.

## 3 RESULTS

The controls and BHD were matched by VO_2_max performances and morphometrically; BHD, though, were taller than controls (Table 1).

Results from arterial and venous blood gases are summarized in Tables 2 and 3.

At rest, BHD and controls had similar blood gas values (Tables 2 and 3).

**TABLE 2:** Venous blood gas parameters of (a) breath‐hold divers (BHD) at rest, after preconditioning, after apnea, and after VO_2_max. (b) controls at rest, after preconditioning, and after VO_2_max.

(a)												
BHD	Rest			After preconditioning			After apnea			After VO_2_max		
Parameter	Native	+Lactate	+Dummy	Native	+Lactate	+Dummy	Native	+Lactate	+Dummy	Native	+Lactate	+Dummy
pH	7.360 ± 0.0413	7.372 ± 0.0533	7.377 ± 0.0526	7.390 ± 0.0182 #	7.394 ± 0.0159	7.394 ± 0.0194	7.393 ± 0.0299 *	7.394 ± 0.0298	7.394 ± 0.0320	7.179 ± 0.0743 (1,2,3*)	7.179 ± 0.0825 (1,2,3*)	7.185 ± 0.0686 (1,2*)
pCO_2_	6.977 ± 1.00	6.74 ± 0.96	6.41 ± 1.16	6.00 ± 1.21	6.07 ± 0.57	5.85 ± 0.90	6.25 ± 0.80	5.82 ± 0.81	5.96 ± 0.92	7.94 ± 2.39 (1,2 ¤)	7.59 ± 2.20	7.89 ± 1.47
pO_2_	5.07 ± 1.39	4.85 ± 1.60	4.85 ± 1.66	4.69 ± 0.74	4.42 ± 0.54	4.75 ± 0.70	3.73 ± 0.89	3.90 ± 1.22	3.81 ± 1.12	4.14 ± 2.75	4.04 ± 3.10	3.36 ± 1.16
Glu	5.3 ± 0.8	5.2 ± 0.8	5.3 ± 0.8	5.0 ± 0.8 #	4.7 ± 0.8	4.8 ± 0.8	4.9 ± 0.5 *	4.9 ± 0.5	4.8 ± 0.5	6.5 ± 0.7 (1,2,3*)	6.4 ± 0.9	6.1 ± 0.7
Lac	1.4 ± 0.2	5.5 ± 2.2 #	1.4 ± 0.3	1.6 ± 0.5	5.7 ± 2.0	1.6 ± 0.4	1.7 ± 0.3 *	6.7 ± 2.3	1.7 ± 0.3	12.0 ± 3.8 (1,2,3*)	17.6 ± 4.5 (1,2,3*)	9.9 ± 2.5 (1,2,3*)
Base exc.	4.1 ± 1.6	3.6 ± 1.2	3.7 ± 1.5 #	3.8 ± 2.1 *	3.3 ± 0.8	2.8 ± 1.9	3.5 ± 2.2 *	1.7 ± 2.3	2.2 ± 2.4	−6.5 ± 5.1 (1,2,3*)	−7.3 ± 5.7 (1,2,3*)	−6.1 ± 3.6 (1,2,3*)
HCO_3_‐	25.7 ± 1.4	25.3 ± 0.9	25.3 ± 1.1	26.2 ± 1.7 *	25.6 ± 0.9	25.4 ± 1.4	25.7 ± 1.3 *	24.5 ± 1.3	24.9 ± 1.2	16.9 ± 3.0 (1,2,3*)	17.3 ± 2.5 (1,2,3*)	17.5 ± 2.2 (1,2,3*)
p50	27.67 ± 1.06	26.82 ± 2.10	26.10 ± 2.71	26.0.97 ± 0.57	26.88 ± 0.46	26.87 ± 0.49	26.83 ± 0.81	26.82 ± 0.94	26.75 ± 0.96	32.03 ± 3.23 (¤1,2,3)	31.46 ± 4.60 (¤1,2,3)	31.98 ± 3.24 (¤1,2,3)

(b)									
Controls	Rest			After preconditioning			After VO_2_max		
Parameter	Native	+Lactate	+Dummy	Native	+Lactate	+Dummy	Native	+Lactate	+Dummy
pH	7.377 ± 0.0171	7.374 ± 0.0182	7.381 ± 0.0194	7.372 ± 0.0119 *	7.369 ± 0.0166	7.375 ± 0.0156	7.205 ± 0.10 (1,2#)	7.166 ± 0.0332 (1, 2¤)	7.166 ± 0.0382 (1, 2¤)
pCO_2_ (kPa)	6.90 ± 0.27	6.49 ± 0.25	6.38 ± 0.32	6.52 ± 0.49	6.29 ± 0.41	6.04 ± 0.48	7.40 ± 1.94	7.023 ± 1.38	7.27 ± 1.73
pO_2_ (kPa)	4.19 ± 1.48	4.21 ± 1.47	4.59 ± 1.74	4.62 ± 0.97	4.91 ± 0.88	5.16 ± 1.04	5.58 ± 3.48	5.86 ± 2.42	5.47 ± 2.76
Glu (mmol/l)	5.5 ± 1.2	5.2 ± 1.2	5.2 ± 1.1	5.5 ± 0.7	5.2 ± 0.6	5.1 ± 0.6	5.1 ± 0.9	5.0 ± 1.0	5.0 ± 0.9
Lac (mmol/l)	1.3 ± 0.23	7.3 ± 1.7	1.2 ± 0.20	1.9 ± 0.20 (1*)	6.6 ± 2.6 ¤	1.8 ± 0.2	11.3 ± 2.1 (1,2*)	17.3 ± 2.5 (1,2,*)	12.3 ± 1.2 (1,2 ¤)
Base exc. (mmol/l)	5.6 ± 1.4	3.6 ± 1.0	3.6 ± 1.5 *	4.1 ± 1.4 #	2.4 ± 1.3	1.8 ± 1.6	−6.5 ± 0.9 (1,2*)	−9.1 ± 2.6 (1,2,*)	−8.5 ± 3.1 (1,2,*)
HCO_3_‐ (mmol/L)	27.0 ± 0.9	25.9 ± 0.6	25.8 ± 1.0 *	26.4 ± 1.1 #	25.2 ± 1.0	24.9 ± 1.2	17.6 ± 1.6 (1,2*)	15.9 ± 1.4 (1,2,*)	16.1 ± 1.6 (1,2,*)
p50 (kPa)	26.82 ± 1.04	26.82 ± 2.10	26.77 ± 1.32	27.03 ± 2.51	26.88 ± 0.46	27.40 ± 1.3	32.15 ± 4.04 (1,2 #)	31.48 ± 4.59 (1,2 ¤)	33.62 ± 2.62 (1,2*)

*Note*: *1: *p* < 0.001 compared to rest. #1: *p* < 0.005 compared to rest. ¤1: *p* < 0.05 compared to rest. *2: *p* < 0.001 compared to preconditioning. #2: *p* < 0.005 compared to preconditioning. ¤2: *p* < 0.05 compared to preconditioning. *3: *p* < 0.001 compared to after maximum apnea. #3: *p* < 0.005 compared to after maximum apnea. ¤3: *p* < 0.05 compared to after maximum apnea.

Abbreviations: Base exc., base excess; Glu, glucose; HCO_3_−, bicarbonate; Lac, lactate; p50, hemoglobin oxygen unloading; pCO_2_, partial pressure of carbon dioxide; pO_2_, partial pressure of oxygen.

**TABLE 3:** Arterial blood gas parameters of (a) controls at rest, after preconditioning, and after VO_2_max (b) breath‐hold divers (BHD) at rest, after preconditioning, after apnea, and after VO_2_max.

(a)									
Controls	Rest			After preconditioning			After VO_2_max		
Parameter	Native	+Lactate	+Dummy	Native	+Lactate	+Dummy	Native	+Lactate	+Dummy
pH	7.462 ± 0.0590	7.467 ± 0.0584	7.425 ± 0.118	7.418 ± 0.0143	7.426 ± 0.0200	7.424 ± 0.0197	7.224 ± 0.0464 (2 *)	7.216 ± 0.040 (2 *)	7.217 ± 0.0481 (2 *)
pCO_2_	4.96 ± 0.93	4.59 ± 0.98	4.89 ± 1.20	5.58 ± 0.43 (¤1)	5.13 ± 0.41 (¤1)	5.24 ± 0.50 ¤1	3.78 ± 0.46 (1,2 #)	3.67 ± 0.49 (2#)	3.69 ± 0.29 (2 #)
pO_2_	13.7 ± 2.4	13.8 ± 2.5	12.9 ± 4.5	11.9 ± 0.9	13.3 ± 1.4	13.2 ± 1.6	15.8 ± 1.5 (1#)	16.1 ± 1.4	15.5 ± 1.4
Glu	6.3 ± 1.2	6.0 ± 1.0	6.1 ± 1.2	5.9 ± 0.8	5.6 ± 0.7	5.7 ± 0.7	5.7 ± 1.3	5.5 ± 1.2	5.4 ± 1.2
Lac	1.0 ± 0.3	6.0 ± 2.6	1.1 ± 0.3	1.1 ± 0.2	6.1 ± 1.3	1.1 ± 0.2	17.8 ± 1.7 (1,2 #)	22.3 ± 1.5 (1,2 #)	16.4 ± 2.0 (1,2 #)
Base exc.	2.3 ± 1.0	0.8 ± 1.5	0.7 ± 1.0	2.5 ± 1.5	1.2 ± 1.9	1.3 ± 1.5	−15.5 ± 2.2 (1,2 #)	−16.2 ± 2.4 (1,2 #)	−15.9 ± 1.8 (1,2 #)
HCO_3_‐	26.6 ± 0.5	25.5 ± 0.8	24.0 ± 3.3	26.3 ± 1.0	25.3 ± 0.8	25.4 ± 1.2	13.8 ± 1.6 (1,2 #)	13.3 ± 1.7 (1,2 #)	13.5 ± 1.5 (1,2 #)
p50	23.2 ± 1.5	22.3 ± 1.02	22.2 ± 2.6	24.7 ± 1.0	24.5 ± 1.2	24.3 ± 2.0	30.0 ± 3.1 (1#)	29.0 ± 2.50 (1#)	30.2 ± 2.9 (1#)

(b)												
BHD	Rest			After preconditioning			After apnea			After VO_2_max		
Parameter	Native	+Lactate	+Dummy	Native	+Lactate	+Dummy	Native	+Lactate	+Dummy	Native	+Lactate	+Dummy
pH	7.447 ± 0.0416	7.452 ± 0.0219	7.453 ± 0.0117	7.437 ± 0.0380	7.444 ± 0.0331	7.441 ± 0.0278	7.375 ± 0.0448 (1,2*)	7.373 ± 0.0415 (1,2*)	7.377 ± 0.0462 (1,2*)	7.238 ± 0.0337 (1,2,3#)	7.238 ± 0.0274 (1,2,3#)	7.235 ± 0.0302 (1,2,3#)
pCO_2_	4.93 ± 0.73	4.60 ± 0.46	4.64 ± 0.47	5.20 ± 0.85	4.80 ± 0.72	5.00 ± 0.67	6.97 ± 0.63 (1,2 #)	6.58 ± 0.59 (1,2*)	6.60 ± 0.78 (1,2*)	3.84 ± 0.55 (1,2,3#)	3.66 ± 0.47 (1,2,3#)	3.64 ± 0.40 (1,2,3#)
pO_2_	14.3 ± 2.2	14.8 ± 2.1	14.1 ± 1.7	13.3 ± 1.9	13.7 ± 1.3	13.9 ± 1.4	4.9 ± 0.5 (1,2*)	5.0 ± 0.7 (1,2*)	5.0 ± 0.4 (1,2*)	15.8 ± 0.7 (1,3* & 2¤)	16.2 ± 0.9 (1,2,3#)	16.4 ± 1.0 (2,3#)
Glu	5.6 ± 0.8	5.4 ± 0.9	5.4 ± 0.9	5.3 ± 0.9	5.1 ± 0.8	5.2 ± 0.8	5.6 ± 0.6	5.5 ± 0.7	5.4 ± 0.7	7.3 ± 1.1 (1,2,3#)	6.5 ± 0.9 (1,2,3#)	6.5 ± 0.8 (1,2,3#)
Lac	1.1 ± 0.1	5.5 ± 1.9	1.1 ± 0.2	1.0 ± 0.3	6.1 ± 2.6	1.0 ± 0.3	1.3 ± 0.3	6.8 ± 1.9	1.2 ± 0.3	18.3 ± 2.0 (1,2,3#)	22.5 ± 3.6 (1,2,3#)	17.3 ± 1.7 (1,2,3#)
Base exc.	1.4 ± 1.8	0.1 ± 1.4	0.5 ± 1.8	1.8 ± 2.2	0.4 ± 1.8	1.0 ± 1.7	4.2 ± 1.7	2.5 ± 1.5	2.7 ± 1.7	−15.1 ± 2.3 (1,2,3*)	−15.8 ± 1.6 (1,2,3#)	−15.9 ± 2.0 (1,2,3#)
HCO_3_‐	25.8 ± 1.2	25.0 ± 1.0	25.2 ± 1.2	26.0 ± 1.4	25.1 ± 1.2	25.5 ± 1.0	25.8 ± 1.7	24.8 ± 1.5	25.0 ± 1.4	14.5 ± 1.3 (1,2,3*)	14.1 ± 0.8 (1,2,3#)	14.0 ± 1.1 (1,2,3#)
p50	23.1 ± 1.8	23.9 ± 2.5	23.4 ± 2.5	23.3 ± 1.7	23.9 ± 1.8 (4*)	23.2 ± 2.0	27.5 ± 2.2 (1,2*)	27.2 ± 1.8 (1,2#)	27.0 ± 2.1 (1,2#)	28.8 ± 1.2 (1,2*)	28.5 ± 1.2 (1,2,3*)	28.6 ± 1.1 (1.2*)

*Note*: *1: *p* < 0.001 compared to rest. #1: *p* < 0.005 compared to rest. ¤1: *p* < 0.05 compared to rest. *2: *p* < 0.001 compared to preconditioning. #2: *p* < 0.005 compared to preconditioning. ¤2: *p* < 0.05 compared to preconditioning. *3: *p* < 0.001 compared to after maximum apnea. #3: *p* < 0.005 compared to after maximum apnea. ¤3: *p* < 0.05 compared to after maximum apnea. *4: *p* = 0.03 compared to native blood samples after preconditioning.

Abbreviations: Base exc., base excess (mmol/L); Glu, glucose (mmol/L); HCO_3_−, bicarbonate (mmol/L); Lac, lactate (mmol/L); p50, oxygen unloading from hemoglobin (kPa); pCO_2_, partial pressure of carbon dioxide (kPa); pO_2_, partial pressure of oxygen (kPa).

**FIGURE 2:**
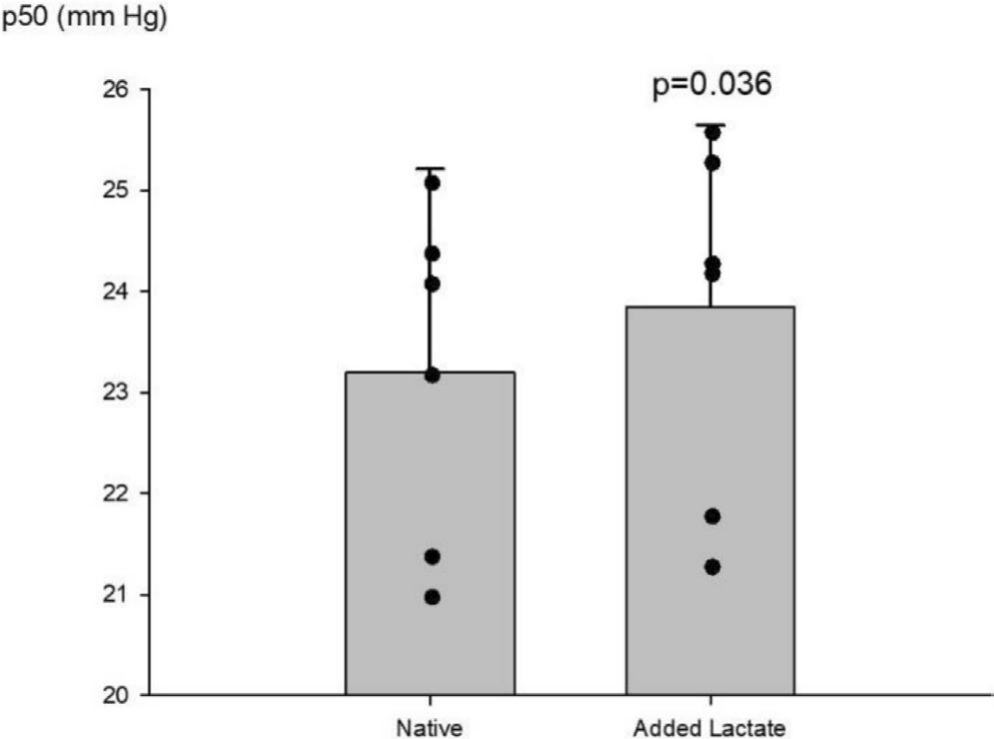
Preconditioned arterial blood of breath‐hold divers. Preconditioned arterial blood from breath‐hold divers. Lactate added in vitro increased p50 (*p* = 0.036). Dots represent individual values.

After RIC, p50 increased (*p* = 0.036) in arterial blood from BHD when adding lactate (Figure 2), whereas this was not the case in controls (Figure 2). Adding lactate to arterial blood samples of BHD also increased glucose after RIC (*p* < 0.05). Similarly, in venous blood samples (from the arm that had RIC), lactate increased after RIC in controls, but not in BHD (*p* = 0.001). Venous blood samples of BHD added placebo solution had lower sO_2_ after RIC as compared to rest (*p* < 0.05), whereas this could not be demonstrated in controls. Adding lactate to the blood samples after preconditioning demonstrated a higher pH in BHD than in the controls (*p* < 0.005). Also, after preconditioning, lactate increased in venous blood samples of controls (*p* < 0.001), but not in BHD.

At end of maximum apnea (BHD only; 375 ± 49 s) arterial pO_2_ decreased from 14.3 ± 2.2 kPa at rest to 4.9 ± 0.5 kPa (*p* < 0.005), while pCO_2_ increased from 4.93 ± 0.73 kPa at rest to 6.97 ± 0.63 kPa and pH decreased from 7.45 ± 0.04 to 7.38 ± 0.045 (*p* < 0.005). Also, p50 increased in arterial blood samples at end of apnea, (*p* < 0.001), but not in venous blood samples (from the arm that had RIC). However, lactate, pH and bicarbonate increased in venous blood samples (*p* < 0.05).

After maximal exercise compared to rest in both groups, arterial pCO_2_, base excess, and pH decreased, while pO_2_, p50, and lactate increased (*p* < 0.005). However, only in arterial blood samples of BHD did glucose increase after maximal exercise (*p* < 0.05) and adding lactate increased glucose further in BHD (*p* < 0.05). Similarly, in venous blood samples (from the arm, that had RIC) after maximal exercise, a decrease in pH, base excess, and bicarbonate could be demonstrated in all subjects compared to rest (*p* < 0.005), while an increase was found in lactate *p* < 0.05. Glucose increased in venous blood samples of BHD after maximal exercise compared to rest (*p* < 0.05) but not in the controls. Venous pH in BHD was higher than in the controls after maximal exercise (*p* = 0.004).

Overall: adding lactate or placebo solutions to blood samples of both controls and BHD decreased bicarbonate (*p* < 0.05; Tables 2 and 3), except after VO_2_max in BHD: bicarbonate did not change.

Lactate dehydrogenase activity of all subjects was within normal ranges. However, BHD had higher lactate dehydrogenase activity 1 (representing the heart and erythrocytes) compared to controls (Table 4, *p* = 0.012).

**TABLE 4:** Lactate dehydrogenase (LDH) fractions in percentage of total activity.

	Breath‐hold divers	Controls
Number of subjects	6 males	6 males
LDH 1 (Heart and erythrocytes)	30.3 ± 3.9*	23.4 ± 3.7
LDH 2 (Reticuloendothelial system and erythrocytes)	28.9 ± 3.0	32.0 ± 1.3
LDH 3 (Lungs)	24.2 ± 5.0	24.2 ± 5.0
LDH 4 (Kidneys)	9.0 ± 0.6	10.8 ± 1.9
LDH 5 (Skeletal muscle and liver)	8.0 ± 1.1	9.5 ± 2.4

*Note*: Values are mean ± standard deviation.

* *p* = 0.012.

## 4 DISCUSSION

The main findings of our study are as follows: (1) BHD had higher cardiac and erythrocyte lactate dehydrogenase activity as compared to controls, and (2) adding lactate in vitro to preconditioned whole blood from BHD increased p50 and hence increased oxygen unloading. This indicates that lactate facilitates oxygen unloading from the erythrocyte by increasing cellular oxygen supply during reperfusion from the preconditioned blood of BHD only, and not in the blood from the controls. (3) The increasing venous lactate in controls after preconditioning, but not in BHD, indicates lactate metabolization in BHD. Similarly, the increasing arterial glucose after apnea after adding lactate also indicates lactate metabolization during RIC, but in BHD only. (4) BHD tolerates critically low arterial saturation indicating extreme tolerance for hypoxia.

Our results suggest that lactate is the substrate metabolically responsible for preconditioning. However, this adaptation could only be demonstrated in BHD. This indicates that individuals highly adapted towards hypoxia may benefit from preconditioning by increasing cellular oxygen supply during reperfusion.

### 4.1 Lactate dehydrogenase activity

The heart of adult harbor seals (Phoca vitulina) possesses the highest lactate dehydrogenase activity compared to terrestrial animals, indicating a reliance on cardiac lactate metabolism during the hypoxic exposure of diving (Fuson et al., 2003). Similarly, in vitro added lactate to whole blood from a freshly hunted and hence preconditioned whale (Balaenoptera acutorostrata) has been demonstrated to increase the amount of oxygen unloaded by as much as 30% (Brix et al., 1990). This indicates an adaptation in the cardiac and erythrocyte metabolism towards hypoxia in diving mammals. Accordingly, our study demonstrates that BHD had ⁓ 30% higher cardiac and erythrocyte lactate dehydrogenase activity as compared to controls.

### 4.2 Oxygen carrying ability of the blood: Erythrocyte metabolism

The erythrocyte metabolizes glucose anaerobically by phosphorylating glucose to form ATP. In contrast to other cells, the glucose in the erythrocyte is catalyzed by phosphoglycerate kinase (Chatzinikolaou et al., 2024). When the erythrocyte's demand for ATP decreases, it catalyzes 1,3‐bisphosphoglycerate with bisphosphoglycerate mutase to 2,3‐bisphosphoglycerate. 2,3‐bisphosphoglycerate binds to the deoxyhemoglobin, which stabilizes and facilitates the release of oxygen in the tissues (Tilton et al., 1991). The erythrocyte does not possess mitochondria, and lactate is the end product released to the extracellular volume from the erythrocyte. Hence, when extracellular lactate increases and simultaneously oxygen unloading, as demonstrated in our study in the arterial blood from preconditioned BHD, we suggest increased (cardiac and) erythrocyte lactate dehydrogenase activity to be the cause. The results from the blood from the highly adapted BHD in our study can be compared to the increased oxygen unloading from the blood from the whale after adding lactate.

The functional properties of hemoglobin from the whale have been demonstrated to be independent of the presence of organic phosphates such as 2,3‐diphospho‐glycerate, but a very small delta H^+^ of oxygen binding, and lactate is an important factor (Giardina et al., 1990). Accordingly, we have previously not found a difference in 2,3‐diphospho‐glycerate levels in BHD as compared to VO_2_max matched controls (Kjeld, Krag, et al., 2024). However, the present study demonstrated that adding lactate to the venous blood samples after RIC demonstrated a higher pH in BHD as compared to controls, and while lactate increased only in venous blood samples of controls, but not in BHD, this suggests that the BHD metabolizes lactate, while the lower pH in BHD after RIC indicates a left shift in the equation:

$${\mathrm{CO}}_{2}+H_{2}O↔H_{2}{\mathrm{CO}}_{3}↔H^{+}+{{\mathrm{HCO}}_{3}}^{-}$$

Thus, the higher pH in BHD after RIC triggers more pronounced non‐phosphorylating conditions and lower leak respiration in the tissues to cope with reactive oxygen species during the hypoxia by dependency on pH and hence a lower level of protons than controls. Accordingly, we have demonstrated that compared to VO_2_max matched controls, the skeletal muscles of BHD are characterized by lower mitochondrial oxygen consumption both during low leak and high (ETS) respiration similar to diving mammals and demonstrating a lower aerobic mitochondrial capacity of the skeletal muscles as an oxygen conserving adaptation during prolonged dives (Kjeld et al., 2018). This indicates that BHDs respond metabolically to preconditioning by increasing oxygen unloading and using protons as facilitators of oxygen release in tissues, by increasing pH after preconditioning from the equation above and causing a more permanent left shift in the equation above.

### 4.3 Remote Ischemic conditioning (RIC) and the diving reflex

Maximal oxygen uptake or exercise capacity during ergometer cycling (de Groot et al., 2010), swimming, ergometer rowing, or dynamic apnea swimming can be increased by RIC (Jean‐St‐Michel et al., 2011; Kjeld et al., 2014). RIC may be achieved by exposing an arm three times to repeated ischemic hypoxia, similarly to what we did in our study. This hypoxic exposure will induce tolerance to ischemia in the rest of the body (Dirnagl et al., 2009). RIC is triggered after three repeated short periods of ischemia separated by corresponding pauses similar to the diving reflex (Bushell et al., 2002; Kjeld et al., 2009), and RIC appears to be cytoprotective, involving transcriptional upregulation of protective pathways in metabolism and ion‐flux, and it preserves mitochondrial membrane integrity and function (Dave et al., 2008; Dirnagl et al., 2009; Endres, Fan, et al., 2000; Endres, Meisel, et al., 2000; Langley et al., 2005). RIC also increases the capacity of BHD for dynamic diving and static breath holds as well as their brain oxygenation during static breath holds (Kjeld et al., 2014). Hence, the mechanism of RIC could be similar to the effect of the diving reflex: the diving reflex is also maximized after the third repeated apnea with short pauses in between (Kjeld et al., 2009), and we therefore suggest the diving reflex and RIC trigger the same physiological phenomenon. The diving reflex probably has a humoral component (Ollenberger et al., 1998), and just like RIC is neurohumorally mediated (Hausenloy et al., 2007; Lim et al., 2010), the diving reflex is mediated via the brainstem (Panneton, Gan, Le, et al., 2012; Panneton, Gan, & Sun, 2012). As mentioned above, elite BHD have similar cardiac and metabolic adaptations as adult diving mammals (Kjeld, Isbrand, et al., 2021; Kjeld, Moller, et al., 2021), and therefore a similar resistance towards oxidative stress as suggested by Joulia et al. (2002). Previous studies have demonstrated RIC to have a positive effect on the performance of elite aerobic athletes as well (Crisafulli et al., 2011; de Groot et al. 2010; Kjeld et al., 2014; Patterson et al., 2015). We suggest that elite aerobic athletes with a higher VO_2_max (de Groot et al., 2010) may benefit from RIC due to an adaptation from repetitive hypoxic exposure (Nielsen et al., 2002) from more intense exercise than the aerobic athletes in the present study with lower VO_2_max.

### 4.4 Lactate as cardiac super fuel in adapted individuals

**FIGURE 3:**
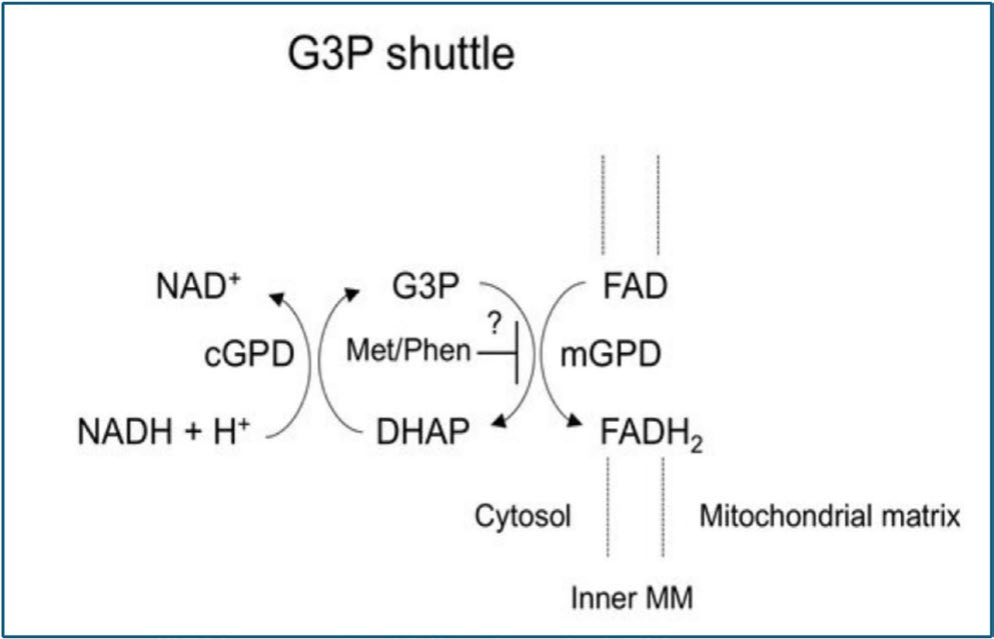
The glycerol‐3‐phosphate (G3P) shuttle. The glycerol‐3‐phosphate shuttle. cGPD, cytosolic glycerol‐3‐phosphate dehydrogenase; mGPD, mitochondrial glycerol‐3‐phosphate dehydrogenase.

Our study was inspired by the in vitro study of Brix et al. (1990), who demonstrated an increased amount of oxygen unloaded by as much as 30% after lactate was added to whole blood from a freshly killed whale (Balaenoptera acutorostrata). The whale was captured and killed after hunting it, where it could be submerged for an average of 37 min (to depths exceeding 200 m) (Christiansen et al., 2015). RIC can be induced following intense exercise (Carvalho de Arruda Veiga et al., 2023), and it can be assumed that the hunting of the diving whale would be exhausting for the animal, akin to the effect of RIC. An important finding of our current study, therefore, is that similar to the study of adding lactate to the blood of freshly killed whales conducted by Brix et al. (1990), our study also demonstrated increased p50 by in vitro adding lactate to the blood of BHD after RIC. This effect was not observed in the control group, and we therefore suggest that only the hypoxia‐adapted BHD would be able to increase oxygen unloading by adding lactate to their blood following RIC. Lactate is the anaerobic metabolic consequence of muscle ischemia (van Hall, 2010), as glucose is enzymatically broken down to pyruvate and in turn lactate in the glycerol‐phosphate shuttle, by transferring electrons from cytoplasmic NADH to the mitochondrial electron transport chain to generate ATP via glycerol‐3‐phosphate (Figure 3) (Philp et al., 2005). Therefore, an upregulation in the glycerol‐phosphate shuttle in the muscles of BHD could be assumed. A similar upregulation of the glycerol‐phosphate shuttle has been observed in ischemic hearts as a compensatory mechanism for energy production (Halestrap, 2010). As discussed below, the upregulation in the glycerol‐phosphate shuttle may be caused by the cardiac ischemia during stable angina pectoris in the population studied by Bøtker et al. and preceding the myocardial infarction (Botker et al., 2010). However, this was not observed in subjects of the study by Hausenloy et al., suggesting that there was no effect of preconditioning (Botker et al., 2010; Hausenloy et al., 2019). This discrepancy might be explained by the adaptations towards hypoxia found in adult diving mammals and BHD, but not in matched aerobic controls and seal pups, indicating that only individuals adapted towards hypoxia can benefit from RIC. Hence, patients with stable angina pectoris prior to myocardial infarction may have had benefit from RIC (Huang et al., 2024), suggesting that the patients in the study by Hausenloy and Botker (2019) were not adapted to hypoxia due to absence of previous stable angina pectoris, and explaining the lack of effect from RIC. Similarly, elite BHDs do not display signs of cardiac ischemia during apneas with SaO_2_ measured as low as 36% (Kjeld, Isbrand, et al., 2024), whether assessed biochemically or by cardiac perfusion imaging (Kjeld et al., 2015; Kjeld, Moller, et al., 2021). In contrast, less well‐adapted BHD have been demonstrated to have both signs of ischemia biochemically and assessed by cardiac imaging (Eichhorn et al., 2018; Kyhl et al., 2016).

In our study, preconditioning elicited less pH response in BHD than controls, and adding lactate to preconditioned blood from BHD increased p50, and hence increased oxygen unloading. Similarly, Brix et al. (1990) found that the effect of oxygen unloading elicited by lactate diminishes when CO_2_ increases, which in turn may increase bicarbonate and hence pH, as in our study. BHD has been demonstrated to possess resistance towards post‐apnoea acidosis and oxidative stress, and the above therefore suggests that only individuals adapted to hypoxia, like diving mammals, high altitude natives, and BHD can benefit from preconditioning (Joulia et al., 2002, 2015). In line with this, our present study demonstrated increasing venous glucose after maximum aerobic exercise in BHD; venous lactate was increasing in controls, but not in BHD after preconditioning, indicating lactate metabolization in BHD as an adaptation towards hypoxia and as previously demonstrated during maximum apnea in BHD (Kjeld, Isbrand, et al., 2021). Lactate and ketone bodies have comparative cardiac metabolic pathways (Dong et al., 2021), and a study by Gormsen et al. (2017) indicates that intravenously administered ketones induce cardiac ketone metabolization and increasing myocardial blood flow, suggestive of ketones being a cardiac super fuel. Cardiac lactate metabolism in diving mammals is 6 times increased as compared to terrestrial animals (Fuson et al., 2003), and as BHD and adult diving mammals share cardiac and metabolic adaptations (Kjeld, Isbrand, et al., 2021; Kjeld, Moller, et al., 2021), we suggest lactate to serve as a cardiac super fuel as well. Since the erythrocyte and the heart share two lactate dehydrogenases (Khan et al., 2020), this may explain the blood‐borne effect of lactate produced by RIC on myocardial ischemia (Wang et al., 2008). Our present results also confirm our previous studies indicating lactate metabolization in elite BHD during maximum apnea (Kjeld, Isbrand, et al., 2021; Kjeld, Moller, et al., 2021), and we suggest that elite BHD can metabolize lactate during intense exercise similar to diving mammals and high altitude populations, but unlike VO_2_max‐matched aerobic athletes (Hochachka et al., 2002; Moraga et al., 2019; van Hall, 2010). This indicates that lactate—instead of being a harmful product of anaerobic metabolism in aerobic athletes (Boning & Schmidt, 2020)—is an important fuel metabolized by mammals adapted to hypoxia like adult diving mammals, high altitude populations, and BHD during maximum exercise.

### 4.5 Limitations

The results of our study were based on a limited number of subjects, since only a few are able to hold their breath for 5 min and even fewer are willing to endure the stress of the protocol.

The p50 was from calculated values. However, our findings in BHD, who share metabolic adaptation to hypoxia with diving mammals, confirms the results found in diving mammals although to a lesser degree.

### 4.6 Perspectives

This study expands the understanding of the extraordinary physiological conditions during sustained voluntary apnea and the adaptations observed in elite BHD, a research area of extreme physiology that remains poorly studied.

Our results support that lactate may increase peripheral oxygenation; however, only in highly adapted individuals, elite BHD. Future studies may reveal whether the adaptations towards hypoxia and erythrocyte and cardiac lactate metabolization found in BHD could optimize performance in other elite athletes and may be of clinical relevance during high‐risk procedures like cardiac surgery. The latter is supported by Leverve et al. (2008), who demonstrated that Ringer lactate prime and infusion compared to colloid result in a better preserved cardiac index during and after cardiac surgery.

## 5 CONCLUSION

In conclusion, we demonstrate that BHDs (1) possess increased cardiac lactate dehydrogenase activity as compared to matched controls, and (2) that adding lactate in vitro to blood from BHD after RIC increases oxygen unloading. (3) Increasing glucose during aerobic exercise in BHD suggests lactate metabolization, as demonstrated previously, and that lactate is fuel for RIC. (4) BHDs tolerate critically low arterial saturation. We suggest that BHDs respond to preconditioning and hypoxia by using lactate in the erythrocyte to increase oxygen unloading and in turn using H+ or protons as facilitators of oxygen release in tissues. RIC and the diving reflex are suggested to be similar physiological phenomena and lactate to be the fuel for both, as an oxygen conserving adaptation in BHDs similar to diving mammals.

## AUTHOR CONTRIBUTIONS

T.K. conceived the study and was in charge of the overall direction and planning of the presented idea and developed the hypothesis. All authors provided feedback, assisted in the analysis, critically revised the manuscript, and approved the final revised version. E.H., T.K., and H.A. contributed to the design of the study. T.K., E.H., T.O.K., J.H., and H.A. carried out the experiments. T.O.K., T.K., E.H., J.H., and H.A. contributed to sample preparation. All authors contributed to the interpretation and analysis of the results.

## FUNDING INFORMATION

This study was not supported by external funding.

## Data Availability

The data that support the findings of this study are available, are all saved and encrypted at hospital servers, but restrictions apply to the availability of these data, which were used under license for the current study, and therefore these data are not publicly available. Data are, however, available from the main author, T.K., upon reasonable request and with permission from Region Hovedstaden & Rigshospitalet.
